# Efficacy and safety of digoxin in acute heart failure triggered by tachyarrhythmia

**DOI:** 10.1111/joim.13565

**Published:** 2022-09-06

**Authors:** Samyut Shrestha, Pedro Lopez‐Ayala, Ibrahim Schaefer, Svetlana S. Nardiello, Androniki Papachristou, Fatima Aliyeva, Cornelia Simmen, Desiree Wussler, Maria Belkin, Danielle M. Gualandro, Christian Puelacher, Eleni Michou, Otmar Pfister, Roland Bingisser, Christian H. Nickel, Tobias Breidthardt, Christian Mueller

**Affiliations:** ^1^ Cardiovascular Research Institute Basel (CRIB) and Department of Cardiology University Hospital Basel University of Basel Basel Switzerland; ^2^ GREAT Network Rome Italy; ^3^ Department of Emergency Medicine University Hospital Basel University of Basel Basel Switzerland; ^4^ Department of Internal Medicine University Hospital Basel University of Basel Basel Switzerland

**Keywords:** acute heart failure, digoxin, tachyarrhythmia

Dear Editor,

Acute heart failure (AHF) is the most common diagnosis in the emergency department (ED) leading to hospitalization [1, 2]. Although tachyarrhythmia is the most common trigger of AHF, the optimal treatment for reducing the heart rate in AHF triggered by tachyarrhythmia is largely unknown. Particular controversy exists regarding the efficacy and safety of digoxin in AHF triggered by tachyarrhythmia [3]. As the negative chronotropic effect of digoxin is mainly thought to be due to increasing vagal tone, few have questioned the possible effectiveness of digoxin in AHF, a situation characterized by substantially increased sympathetic tone [4]. On the other hand, among all currently available negative chronotropic agents, digoxin is the only one with positive inotropic effects, and it is inexpensive and widely available, rendering digoxin a very attractive option. Therefore, the aim of this study was to evaluate the short‐term efficacy and safety of digoxin loading in consecutive patients presenting with AHF triggered by tachyarrhythmia.

This was a single‐center, retrospective cohort study including patients from January 2012 until December 2018 admitted to the University Hospital Basel, with AHF triggered by tachyarrhythmia (atrial fibrillation/atrial flutter). For this analysis, patients were eligible if they were naïve to digoxin (or digitoxin) and received at least 0.75 mg i.v. or per os (the minimal expected effective dose) digoxin for heart rate control in either the ED, the general medical ward, or the intensive care unit (ICU) [5]. Medical data of interest, including vital signs, laboratory values, medications prior to and after digoxin use, and predefined adverse events (AEs) possibly related to digoxin were obtained from the detailed medical chart (Online Supplement). Beta blocker was converted to metoprolol‐equivalent dose for calculation [6]. The study was approved by the ethics committee (reference number: 2019‐00956) for all the patients that had not refused the general informed consent for the use of routinely collected data.

The primary efficacy outcome was heart rate reduction at 24 h and 48 h after 0.75 mg or more of digoxin was given. The primary safety outcomes were AEs possibly related to digoxin, transfer to the ICU, and those observed compared to predicted 30‐day mortality. MEESSI score was calculated to compare observed and predicted mortality [7]. Secondary endpoints included change in systolic and diastolic blood pressure at 24 h and 48 h.

Among 210 patients recruited between January 2012 and December 2018, the mean age was 79 years, 61% were women, and most had mild‐to‐moderate renal dysfunction (Table S1). Twenty‐three percent of patients had received intravenous beta blockers in the ED prior to receiving digoxin. No patient received additional intravenous beta blocker within 48 h of digoxin (Table S2). One hundred and fifty‐seven patients (75%) remained in atrial fibrillation 48 h after receiving at least 0.75 mg of digoxin. Ninety‐one patients (43%) were discharged with the maintenance dose of digoxin. One hundred and twenty‐two patients (58%) received at least 0.75 mg digoxin within 24 h of admission, while 26% received it within 48 h and the rest of the patients received it after 48 h of admission. Among these patients, heart rate was significantly reduced from 141 ± 22 beats per minute (bpm) at presentation to 101 ± 23 bpm at 24 h and 98 ± 22 bpm at 48h (both *p* < 0.001). Systolic blood pressure increased significantly after 24 h and 48 h (*p* < 0.001) with a consistent response irrespective of sex or renal function (Fig. 1, Tables S3 and S4). One patient (0.5%) experienced relevant nausea possibly related to digoxin (Table S5). Predicted and observed 30‐day mortality (10.9% [95% confidence Interval (CI): 9.4–12.5] and 9.0% [95% CI 5.9–13.7], respectively) were comparable.

**Fig. 1:**
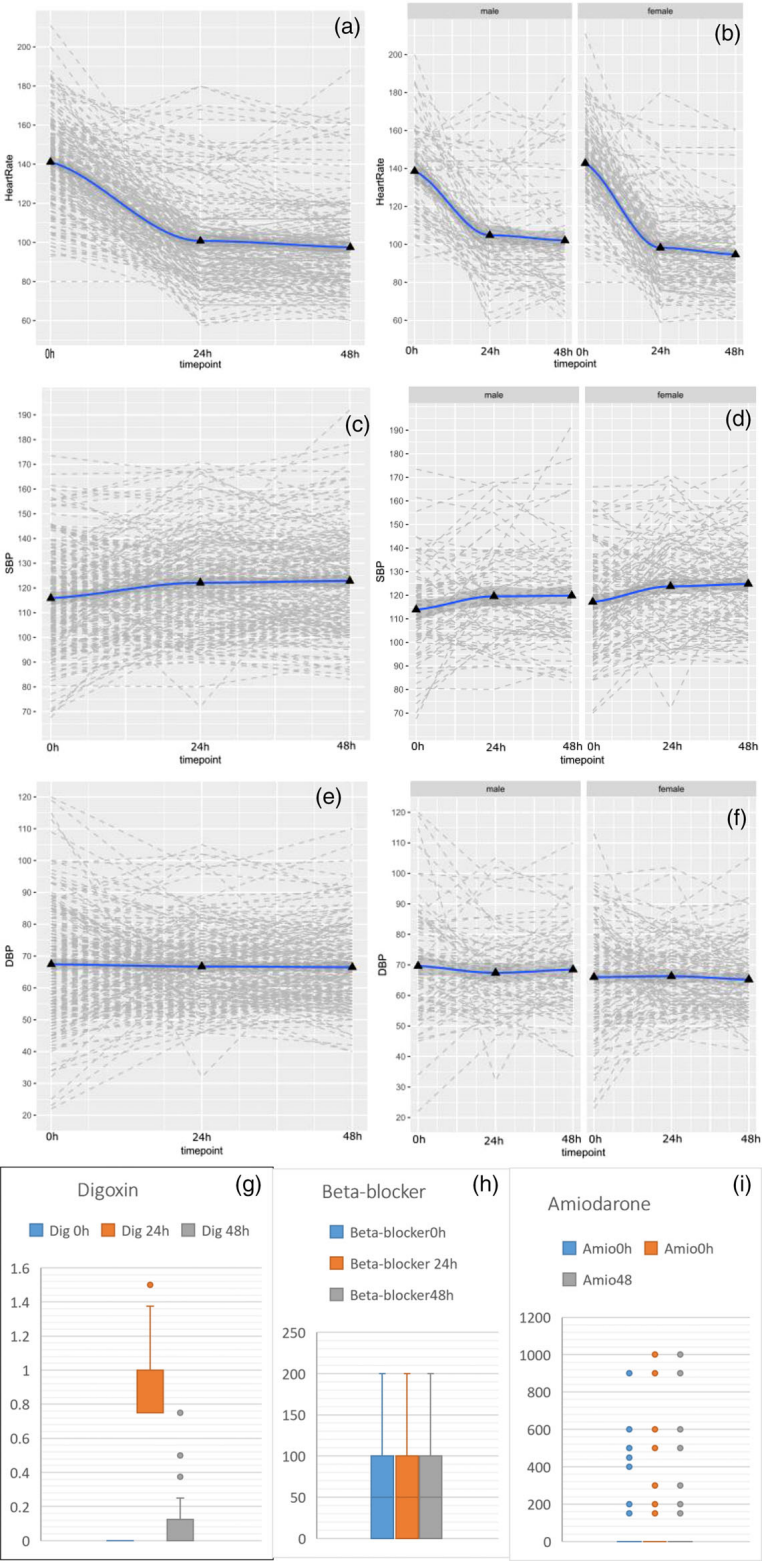
Efficacy of digoxin. Panels A and B show changes in heart rate from 0 (baseline) to 24 h and 48 h after receiving digoxin (at least 0.75 mg) in all patients and stratified by sex, respectively. Panels C and D show changes in systolic blood pressure (SBP) from baseline to 24 h and 48 h after receiving digoxin (at least 0.75 mg) in all patients and stratified by sex, respectively. Panels E and F show changes in diastolic blood pressure (DBP) from baseline to 24 h and 48 h after receiving digoxin (at least 0.75 mg) in all patients and stratified by sex, respectively. The solid line indicates the mean. Panels G, H, and I show the median dose, interquartile range, and 25% and 75% percentiles of digoxin, beta blocker (metoprolol equivalent dose), and amiodarone at 0, 24, and 48 h, respectively. In panel I, the median dose, interquartile range, and 25% and 75% percentiles of amiodarone at 0, 24, and 48 h are all zero (0). The dots represent the outliers.

These findings extend and corroborate observations from two propensity score‐matched studies regarding long‐term outcomes in patients hospitalized for AHF and atrial fibrillation in the United States.

Digoxin initiation was associated with a lower risk of AHF readmission and not with mortality [8, 9].

These findings also seem to support the safety of an unadjusted loading dose in elderly patients with mild‐to‐moderate renal dysfunction, although this was not formally tested.

Some limitations warrant consideration when interpreting the findings of this study. First, this was a single‐center study. Second, this was an uncontrolled study and co‐administration of other negative chronotropic drugs after digoxin loading could have confounded the effect of digoxin. Therefore, it is important to highlight that no relevant changes in the doses of the beta blocker occurred and less than 10% of patients received additional doses of amiodarone within the 48 h study period.

In conclusion, digoxin was very effective in reducing the heart rate among patients with AHF triggered by tachyarrhythmia with a favorable safety profile. It offers physicians a widely available and inexpensive treatment option for these patients.

## Funding

The study was supported by research grants from the University Hospital Basel, the University of Basel, the Swiss National Science Foundation, the Swiss Heart Foundation, the Kommission für Technologie und Innovation (KTI), Abbott, Beckman Coulter, Ortho Clinical Diagnostics, Quidel, Roche, Siemens, and Singulex.

## Conflict of interest

Christian Mueller has received research support from the Swiss National Science Foundation, the Swiss Heart Foundation, the KTI, the European Union, the University of Basel, the University Hospital Basel, Abbott, Beckman Coulter, Idorsia, Ortho Clinical Diagnostics, Quidel, Roche, Siemens, Singulex, and Sphingotec as well as speaker honoraria/consulting honoraria from Acon, Amgen, Astra Zeneca, Boehringer Ingelheim, BMS, Idorsia, Novartis, Osler, Roche, and Sanofi outside of the submitted work. Danielle M. Gualandro received research grants from FAPESP (Fundacao de Amparo a Pesquisa do Estado de Sao Paulo, Brazil) and consulting honoraria from Roche, outside the submitted work. Pedro Lopez‐Ayala has received research support from the Swiss Heart Foundation (FF20079). Tobias Breidthardt received speaker or advisory fees from AstraZeneca, Daiichi‐Sankyo, Roche, and Vifor. These payments were made directly to the University Hospital Basel and no personal payments were received. Christian Puelacher reports research funding from Roche Diagnostics, the University of Basel, and the University Hospital Basel, outside of the submitted work. All other authors declare that they have no conflict of interest with this study.

## Disclosure

The authors designed the study, gathered and analyzed the data, vouch for the data and analysis, wrote the letter, and decided to publish. Christian Mueller, Pedro Lopez‐Ayala, Fatima Aliyeva, Androniki Papachristou, and Samyut Shrestha had full access to all the data in the study and take responsibility for the integrity of the data and the accuracy of the data analysis. All authors have read and approved the letter. The letter and its contents have not been published previously and are not being considered for publications elsewhere in whole or in part in any language, including publicly accessible websites or e‐print servers.

## Supporting information

Supplementary Material
