# Supplementary material for: Efficacy and safety of digoxin in acute heart failure triggered by tachyarrhythmia
Source: J Intern Med. 2022 Sep 6;292(6):969–72. doi: 10.1111/joim.13565 (PMC9826082; doi:10.1111/joim.13565)
Supplement: Supplementary file 1 — Supplementary Material [file JOIM-292-969-s001.docx]

**Online (Supplement)**

Data were collected and analyzed according to the STROBE (strengthening the reporting of observational studies in epidemiology) guidelines. Continuous variables are presented as mean (standard deviation [SD]) or median (interquartile range [IQR]); categorical variables as numbers and percentages. T-test or Mann-Whitney-U test were used for comparing continuous variables, and Pearson’s Chi-square test or Fischer’s Exact test for categorical variables, when appropriate. One-way repeated measure ANOVA was used to determine if there were differences in heart rate and blood pressure before digoxin use, 24h and 48h afterwards. The MEESSI score was calculated on admission to compare the 30-days observed and predicted mortality. As a prior study suggested sex-differences in the risk-benefit ratio of digoxin, subgroup analyses according to sex were predefined. All hypothesis testing was two-tailed and p-values <0.05 were considered statistically significant. The 95% confidence intervals (CIs) were calculated using Wilson’s method for the observed and predicted mortality. All statistical analyses were performed using Stata, version 16.1 (Stata Corp, College Station, TX, USA) and R, version 3.6.3 (R foundation for Statistical Computing).

Heart rate was taken from ECG if available. If no ECG was available, pulse rate was taken. The fastest heart rate before the digoxin administration was taken as the baseline heart rate. The second heart rate (24h heart rate) was the fastest heart rate between 24 hours to 48 hours after digoxin and the third heart rate (48h heart rate) was the fastest heart rate from 48 hours till 72 hours after digoxin. The baseline blood pressure was taken as the average blood pressure between the first blood pressure and the lowest blood pressure before the digoxin administration. Second blood pressure was the average blood pressure taken between 24 hours and 48 hours. Third blood pressure was the average blood pressure taken between 48 hours and 72 hours. All medical data of interest were obtained from the detailed medical chart review of doctors as well as the detailed hospital records documented by the nurse.

**Online Table 1:** Baseline characteristics

|  | **All patients** | **Alive** | **Dead** | **"p value"** |
| --- | --- | --- | --- | --- |
|  | n=210 | n=191 | n=19 |  |
| **Sociodemographic findings** |  |  |  |  |
| Age (years) (mean [SD]) | 78.9 (9.8) | 78.7 (9.7) | 80.8 (11.2) | 0.36 |
| Sex Female (%) | 128 (61) | 118 (61.8%) | 10 (52.6%) | 0.44 |
| **Medical history** |  |  |  |  |
| Heart Failure (%) | 112 (53) | 104 (54.5) | 8 (42.1) | 0.34 |
| CAD (%) | 59 (28) | 55 (28.8%) | 4 (21.1%) | 0.60 |
| History of MI (%) | 26 (12) | 25 (13.1%) | 1 (5.3%) | 0.48 |
| LVEF (median [IQR]) | 45.0 [33.0, 55.0] | 45.0 (32.0, 55.0) | 47.5 (39.0, 65.0) | 0.21 |
| Valvular Heart Disease (%) | 117 (59) | 110 (60.4%) | 7 (41.2%) | 0.13 |
| Active cancer | 27 (13) | 21 (11.0%) | 6 (31.6%) | 0.011 |
| COPD | 31 (14.8%) | 28 (14.7%) | 3 (15.8%) | 1.00 |
| **Devices** |  |  |  | 0.548 |
| No devices | 194 (92) | 177 (93) | 17 (89) |  |
| ICD/CRT | 5 (2) | 5 (3) | 0 (0) |  |
| Pacemaker | 11 (5) | 9 (5) | 2 (11) |  |
| **Chronic Medication** |  |  |  |  |
| Beta-blocker (%) | 129 (61.4) | 120 (62.8%) | 9 (47.4%) | 0.19 |
| Amiodarone (%) | 8 (3.8) | 7 (3.7%) | 1 (5.3%) | 0.54 |
| OAC/NOAC (%) | 110 (52.4) | 103 (53.9%) | 7 (36.8%) | 0.16 |
| ACE-inhibitor/ARB (%) | 100 (47.6%) | 94 (49.2%) | 6(31.6%) | 0.14 |
| Mineralocorticoid receptor blocker (%) | 32 (15.2%) | 29 (15.2%) | 3 15.38%) | 1.00 |
| **Laboratory values** (median [IQR]) |  |  |  |  |
| Potassium, mmol/L | 4.2(3.8, 4.6) | 4.2 (3.8, 4.6) | 4.5 (4.0, 5.3) | 0.028 |
| Creatinine, µmol/L | 98.0 (81.0, 128.0) | 98.0 (81.0, 127.0) | 96.0 (67.0, 148.0) | 0.87 |
| GFR (CKD-EPI) ml/min/1.73m2 | 57.7 (41.2, 74.4)) | 53.5 (39.0, 70.0) | 55.9 (32.4, 80.4) | 0.72 |
| CRP, mg/l | 18.7 (5.9, 58.4) | 17.4 (5.7, 51.9) | 109.0 (9.8, 250.5) | 0.003 |
| Hemoglobin, g/L | 128.0 (111.0, 139.0) | 128.0 (112.0, 139.0) | 116.0 (96.0, 127.0) | 0.003 |
| **Sodium,** mmol/L | 139.0 (135.0, 141.0) | 139.0 (135.0, 141.0) | 138.0 (135.0, 144.0) | 0.71 |
| hs- cardiac troponin T, ng/L | 34.0 (23.0, 53.0) | 33.0 (22.0, 51.0) | 60.0 (33.0, 108.0) | 0.072 |

ACE-inhibitor= Angiotensin converting enzyme inhibitor; ARB= Angiotensin receptor blocker; CAD= coronary artery disease; COPD= chronic obstructive pulmonary disease; CRP= C - reactive protein; CRT= cardiac resynchronization therapy; GFR (CKD-EPI) = Glomerular filtration rate (according to chronic kidney disease epidemiology collaboration) ; hs: High-sensitive; ICD: Implantable cardioverter defibrillator; IQR=Interquartile range; IV= intravenous; LVEF= left ventricular ejection fraction(%); MI= myocardial infarction; NOAC= Novel anticoagulants; OAC= Oral anticoagulants; Valvular Heart Disease= Moderately Severe-Severe aortic/tricuspid, mitral valve stenosis or insufficiency

**Online Table 2. Amount of dose of digoxin, betablocker and amiodarone at 0 hours, 24 hours and 48 hours**

|  | 0h | 24h Dose | 48h Dose |
| --- | --- | --- | --- |
| Digoxin |  | n=211, Median 0.75 (IQR: 0.75, 1) | n=211, Median 0.125 (IQR: 0,0.125) |
| Betablocker (Metoprolol) | n=145, Median 50(IQR 0,100 ) | n= 155 , Median 50 (IQR 0,100) | n= 155 , Median 50 (IQR 0,100) |
| Amiodarone | n=10, Median 0(IQR : 0,0 ) | n=7, Median 0(IQR : 0,0 ) | n=7, Median 0(IQR : 0,0 ) |

h= hours

**Online Table 3. Mean Heart rate and systolic blood pressure in all patients and stratified by sex**

| Overall | | | Male | | | Female | | |
| --- | --- | --- | --- | --- | --- | --- | --- | --- |
| SBP 0h | SBP 24h | SBP 48h | SBP (0h) | SBP (24h) | SBP (48h) | SBP (0h) | SBP (24h) | SBP (48h) |
| 115  (SD:19.8) | 122    (SD:19) | 122  (SD:19.9) | 113  (SD:19.8) | 119    (SD:19.1) | 119  (SD:21) | 117  (SD:119.7) | 123  (SD:18.8) | 124  (SD:17.8) |
| HR 0h | HR 24h | HR 48h | HR0h | HR 24h | HR 48h | HR 0H | HR 24h | HR 48h |
| 141  (SD:21.7) | 100    (SD:22.7) | 97.5  (SD:22.4) | 138  (SD 21.8) | 104  (SD: 24.7) | 102  (SD:26.0) | 142  (SD: 21.6) | 98.21  (SD:21) | 94  (SD:19.3) |

h= hours, HR= heart rate, SBP= systolic blood pressure

**Online Table 4. Mean Heart rate and systolic blood pressure in all patients stratified by GFR**

| GFR <60 | | | GFR ≥60 | | |
| --- | --- | --- | --- | --- | --- |
| SBP 0h | SBP 24h | SBP 48h | SBP (0h) | SBP (24h) | SBP (48h) |
| 112.96    (SD:18.36) | 121.69  (SD:17.38) | 1221.58  (SD:17.46) | 119 .72  (SD:21.11) | 122.66    (SD:21.14) | 124.54  (SD:21.42) |
| HR 0h | HR 24h | HR 48h | HR0h | HR 24h | HR 48h |
| 137  (SD:21.66) | 99  (SD:23.39) | 97  (SD:22.25) | 145  (SD 21.16) | 102  (SD: 21.86) | 97  (SD:22.82) |

h= hours, HR= heart rate, SBP= systolic blood pressure

**Online Table 5. Adverse effects in all patients**

| N | 210 |
| --- | --- |
| 30-day death | 19 (9%) |
| ICU referral after digoxin | 6(3%) |
| Others (nausea, vomiting, visual impairment) | 1 (0.5%) |
